# Nanobioarchitectures based on chlorophyll photopigment, artificial lipid bilayers and carbon nanotubes

**DOI:** 10.3762/bjnano.5.240

**Published:** 2014-12-02

**Authors:** Marcela Elisabeta Barbinta-Patrascu, Stefan Marian Iordache, Ana Maria Iordache, Nicoleta Badea, Camelia Ungureanu

**Affiliations:** 1 Department of Electricity and Magnetism, Solid-State Physics and Biophysics, Faculty of Physics, University of Bucharest, 405 Atomistilor Street, P.O. Box MG-11, Bucharest-Magurele, 077125, Romania; 2 3Nano-SAE Research Centre, Faculty of Physics, University of Bucharest, P.O. Box MG-38, Bucharest-Magurele, 077125, Romania; 3 Department of General Chemistry, Faculty of Applied Chemistry and Materials Science, University Politehnica of Bucharest, 1–7 Polizu Street, 011061, Bucharest, Romania

**Keywords:** antibacterial activity, antioxidant properties, artificial lipid bilayers, carbon nanotubes, chlorophyll

## Abstract

In the last decade, building biohybrid materials has gained considerable interest in the field of nanotechnology. This paper describes an original design for bionanoarchitectures with interesting properties and potential bioapplications. Multilamellar lipid vesicles (obtained by hydration of a dipalmitoyl phosphatidylcholine thin film) with and without cholesterol were labelled with a natural photopigment (chlorophyll *a*), which functioned as a sensor to detect modifications in the artificial lipid bilayers. These biomimetic membranes were used to build non-covalent structures with single-walled carbon nanotubes. Different biophysical methods were employed to characterize these biohybrids such as: UV–vis absorption and emission spectroscopy, zeta potential measurements, AFM and chemiluminescence techniques. The designed, carbon-based biohybrids exhibited good physical stability, good antioxidant and antimicrobial properties, and could be used as biocoating materials. As compared to the cholesterol-free samples, the cholesterol-containing hybrid structures demonstrated better stability (i.e., their zeta potential reached the value of −36.4 mV), more pronounced oxygen radical scavenging ability (affording an antioxidant activity of 73.25%) and enhanced biocidal ability, offering inhibition zones of 12.4, 11.3 and 10.2 mm in diameter, against *Escherichia coli, Staphylococcus aureus* and *Enterococcus faecalis*, respectively.

## Introduction

The “nanoworld” has long fascinated scientists due to the interesting properties that small-dimensional objects provide (as compared to their bulk counterparts) and the design of bionanohybrids has gained considerable interest in the fields of nanotechnology and biomedicine [1–3]. Special attention has been paid to biomimetic membranes that convey biocompatibility to the hybrid materials [4–7].

One of the building blocks used to construct nanomaterials are carbon nanotubes (CNTs), which are allotropes of carbon with a unique nanostructure consisting of graphene sheets (layers of sp^2^-hybridized carbon atoms, perfectly hexagonally packed in a honeycomb network) rolled up into tubular shapes with a very large length/diameter ratio. This structure gives the unusual properties of CNTs such as: high mechanical strength (due to C–C sp^2^ bonds, which is one of the strongest bonds), flexibility without breakage or damage, high elasticity, good electrical conductivity, and chemical stability. These cylindrical graphene nanotubes are considered one of the most attractive nanomaterials. Applicability of CNTs in the biomedical field is complicated as they are completely insoluble in all solvents and are present as bundles. Thus, they have a tendency to aggregate due to van der Waals forces, π–π stacking and hydrophobic interactions among individual CNTs, making them difficult for characterization, handling, and analytical investigations. These problems can be overcome by functionalization [8–9].

Carbon nanotubes are widely used in the biomedical field due to their unusual properties and because CNT toxicity occurs only in the pristine form and in very high doses [10–11]. Kam et al. [12] observed functionalized single-walled carbon nanotubes (f-SWCNTs, 0.05 mg/mL) internalized inside hamster ovary cells without toxicity. Immature dendritic cells incubated with doses of SWCNTs up to 100 μg/mL had no effect on their functionality and viability [13]. Many scientific papers report that oral administration of both pristine and f-CNTs do not induce toxicity in mice [14–17].

On the other hand, biocoating SWCNTs with biomolecules such as phospholipids conveys biocompatibility and less toxicity to carbon nanotubes. Moreover, SWCNTs are characterized by less accumulation in body as compared to multi-walled carbon nanotubes [18]. Bianco et al. [19] showed that carbon nanotube biofunctionalization lead not only to the improved solubility and biocompatibility of CNTs, but also transformed them into platforms for biomedical applications. Carbon nanotubes are generally considered biocompatible and of low toxicity for biomedical purposes. To this regard, Firme and Bandaru [11] reviewed the applications of carbon nanotubes to biological systems and highlighted the possibility that cells have cascade reactions that can resist the toxicity induced by CNTs. Ghafari et al. [20] observed that CNTs can be actively ingested and excreted from cells without any observable toxicity effects (e.g., as in case of *Tetrahymena thermophila* bacteria). Furthermore, Kagan et al. [21] pointed out that certain enzymes such as myeloperoxidases can degrade carbon nanotubes, breaking them down into water and carbon dioxide.

CNTs are largely used as drug delivery vehicles, showing potential in targeting specific cancer cells [18] with a necessary dosage lower than conventional drugs, without harming healthy cells and significantly reduced side effects. Another interesting property of carbon nanotubes is their antioxidant activity, which has been exploited in the preparation of anti-aging cosmetics and sunscreen creams to protect skin against free radicals formed by the body or by UV sunlight [10].

The goal of this work is to achieve antioxidant and antibacterial bionanomaterials based on liposomes and carbon nanotubes, which could open new perspectives for biomedical and biotechnological applications. The increased interest in use of phospholipids is due to the fact that they are basic structural components of biomembranes and artificial lipid membranes (liposomes). Liposomes are spherical, soft-matter vesicles composed of one or more lipid membranes (called lamellae) separated by aqueous compartments [22], with the structure of their lipid bilayers resembling that of cell membranes.

In this work, we present the preparation of complex biocomposites based on liposomes and carbon nanotubes. Chlorophyll *a* is used as a molecular sensor (or as a spectral marker in spectroscopic methods) for rapid monitoring of the preparation of the complex biohybrid materials, which provides evidence of the interaction of CNTs with the versatile models of biomembranes for possible biomedical applications. This work encompasses the research stage with the design, preparation and characterization techniques needed for monitoring these biomaterials, and presents new interdisciplinary aspects involving concepts of biochemistry, biophysics, microbiology, nanotechnology, colloid and supramolecular chemistry, and materials science. The biophysical studies on interaction between the nanostructures and amphiphilic molecules presented here allow for an understanding the structure of molecular assemblies and facilitate the full exploitation of the bioapplicability potential of the resulting bionanomaterials.

## Experimental

### Materials

Luminol (5-amino-2,3-dihydro-1,4-phthalazinedione), KH_2_PO_4_, Na_2_HPO_4_, Tris (tris(hydroxymethyl)aminomethane), HCl, and H_2_O_2_were purchased from Merck (Germany). Methanol (99.9%), SWCNTs and the lipids used for the liposome preparation (dipalmitoylphosphatidylcholine, DPPC and cholesterol, Chol) were supplied from Sigma-Aldrich (Germany).

The antimicrobial activity was tested against human pathogenic bacteria such as *Staphylococcus aureus* ATTC 25923, *Escherichia coli* ATCC 8738, and *Enterococcus faecalis* ATCC 29212. The bacterial strains were grown in Luria Bertani Agar (LBA) plates at 37 °C with the following composition: peptone (Merck, 10 g/L), yeast extract (Biolife, 5 g/L), NaCl (Sigma-Aldrich, 5 g/L) and agar (Fluka, 20 g/L).

### Synthesis

#### Liposome preparation

The hydration method [22] of a thin DPPC film was used to obtain two kinds of multilamellar lipid vesicles (MLVs, 0.5 mM) with and without cholesterol in the artificial lipid bilayers (DPPC:Chol molar ratio = 4:1), which were suspended in a safe bio-dispersant of phosphate buffer solution (PB, KH_2_PO_4_–Na_2_HPO_4_, pH 7.4). Chlorophyll *a* (Chl*a*), a natural antioxidant porphyrin, was extracted from spinach leaves according to the Strain and Svec method [23]. Considering its antioxidant properties [24–25] and spectral features, this photopigment was chosen and inserted (Chl*a*:lipid molar ratio = 1:100) into the both types of liposomes: Chl*a*–DPPC–MLVs (sample V1) and Chl*a*–Chol (20%)–DPPC–MLVs (sample V2) as described previously [4–6]. A summary of the sample abbreviations used in this work are presented in Table 1.

**Table 1:** The sample composition and abbreviations for the biostructures prepared in this work.

Sample description	Sample name

Chl*a*–DPPC–MLVs	V1
Chl*a*–Chol–DPPC–MLVs	V2
Chl*a*–DPPC–MLVs/CNTs hybrid	V3
Chl*a*–Chol–DPPC–MLVs/CNTs hybrid	V4

#### Preparation of the liposome/SWCNT biocomposites

**Figure 1:**
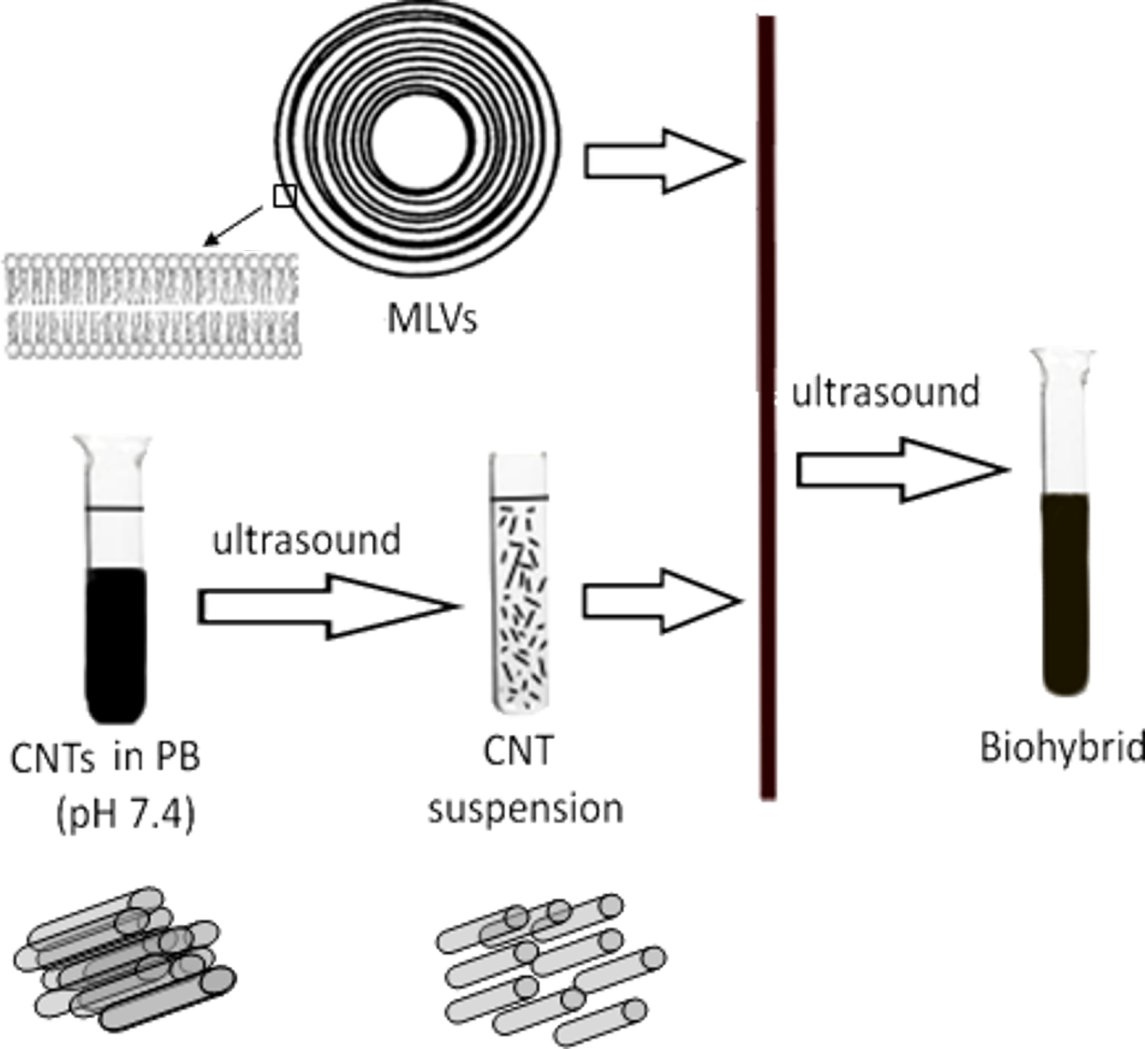
Schematic representation of the biohybrid preparation.

Small aliquots of a previously sonicated SWCNT stock suspension (0.9 mg/mL, in PB pH 7.4) were added to a liposome suspension and the resulting mixture was subjected to ultrasound treatment (Hielser titanium probe sonicator, UP 100H, 15 min with breaks). Figure 1 shows the schematic representation of the ultrasound-mediated biohybrid preparation resulting in two types of biocomposites: Chl*a*–DPPC–MLVs/CNTs hybrid (sample V3) and Chl*a*–Chol–DPPC–MLVs/CNTs hybrid (sample V4). Due to the photosensitivity of the samples, all the experiments were carried out in dark.

### Characterization methods

#### Absorption spectroscopy

The absorption spectra were recorded using a double beam UV–vis spectrophotometer (M400, Carl Zeiss, Germany) in range of 200–800 nm, with a resolution of 1 nm using a 1 nm slit width and a 0.3 nm/s scan rate.

#### Fluorescence analysis

The fluorescence emission spectra of Chl*a* in liposomes and hybrid structures were collected on a Perkin-Elmer, LS55 fluorescence spectrometer. The samples were excited with 430 nm excitation light.

Fluorescence anisotropy measurements were carried out on the same spectrofluorometer (using parallel and perpendicular polarizers) fitted with a biokinetic accessory, affording continuous monitoring of the temperature in the cuvette and magnetic stirring of the suspensions of liposomes and biohybrids. Slit widths of 7.5 and 4 nm were used for the excitation and emission window, respectively. The anisotropy was calculated as the mean value of seven independent measurements, at a specified temperature, using the equation:

[1]
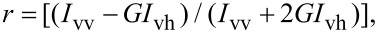


where *I* represents the relative fluorescence intensity, the subscripts v and h represent the vertical and horizontal orientation of the excitation and emission polarizers, respectively, and *G*, the instrumental grating factor, is defined as the ratio of the sensitivities of the detection system for vertically and horizontally polarized light [26].

#### Zeta potential determination

The measurement of the electrokinetic potential is used to assess the charge stability of a disperse system. The measurement of the zeta potential (ZP) was realized by use of a Zetasizer Nano ZS (Malvern Instruments Ltd., U.K.). The ZP is measured by applying an electric field across the analyzed aqueous dispersion. All measurements were performed in triplicate.

#### Dynamic light scattering (DLS) measurements

The size of the liposomes represented by the hydrodynamic diameter, *Z*_average_ (the particle diameter plus the double layer thickness) was measured using DLS (Zetasizer Nano ZS, Malvern Instruments Ltd., U.K.) at 25 °C at a scattering angle of 90°. The average diameters (based on the Stokes–Einstein equation) and the polydispersity index (PDI, indicating the width of the size distribution) were obtained from 3 individual measurements using an intensity distribution.

#### Atomic force microscopy (AFM)

AFM images were recorded on an integrated platform, AFM/SPM (NTEGRA Prima, NT-MDT, USA) in semi-contact mode (scanning area range 1.2 × 1.2 µm^2^) using an NSG01 cantilever with a typical radius of curvature of 10 nm. All AFM measurements were obtained on samples deposited on Si plate substrates.

#### Chemiluminescence (CL) assay

The in vitro antioxidant activity of the samples was determined by chemiluminescence (CL) assay using a chemiluminometer (Turner Design, TD 20/20, USA). A wide range of oxygen free radicals (reactive oxygen species, ROS) [27–28] was formed by a generator system based on H_2_O_2_ in an alkaline buffer solution (Tris·HCl, pH 8.6) mimicking an oxidative stress. Luminol was introduced as light amplifier in this system in order to increase the detection sensitivity of activated oxygen species. The antioxidant activity (*AA*, %) was calculated as a percentage of free radical scavenging of each sample using:

[2]
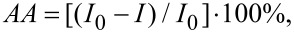


where *I*_0_ is the maximum CL intensity for a standard sample at *t* = 5 s, and *I* is the maximum CL intensity for a sample at *t* = 5 s [29]. Three measurements were performed for each sample in order to accurately evaluate the antioxidant activity.

#### Antibacterial assay

The antibacterial activity of the samples was tested against Gram-positive and Gram-negative bacteria from the American Type Culture Collection (ATCC). In the present study three bacterial strains were used for the antibacterial assay: *Staphylococcus aureus* (ATCC 6538), *Enterococcus faecalis* (ATCC 29212) and *Escherichia coli* (ATCC 8738). The microorganisms used in this study were selected because of their clinical importance in terms of medical and food applications [30–35]. The bacterial strains were cultivated in a tube containing a Luria Bertani (LB) medium as reported recently by Ansari et al. [36]. The Kirby–Bauer disk-diffusion method was performed to determine the antibacterial potential of the samples [37]. Sterile LBA plates were prepared by pouring the sterilized media into sterile Petri plates (diameter = 90 mm) under aseptic conditions.

The sensitivity of the microorganism species to the biohybrids prepared was determined by measuring the size of inhibitory zones (including the diameter of sample) on the agar surface around the sample with a minimum cut-off value set at 8 mm. The inhibition zone was measured and expressed in millimetres. In this study triplicate plates were prepared for each sample and bacterial strain. The mean zone of inhibition was calculated with a standard deviation procedure. The data were presented as mean ± standard deviation (SD). The SD was calculated as the square root of variance using the STDEV function in Excel 2010.

## Results and Discussion

### Spectral characterization of nanobioarchitectures

**Figure 2:**
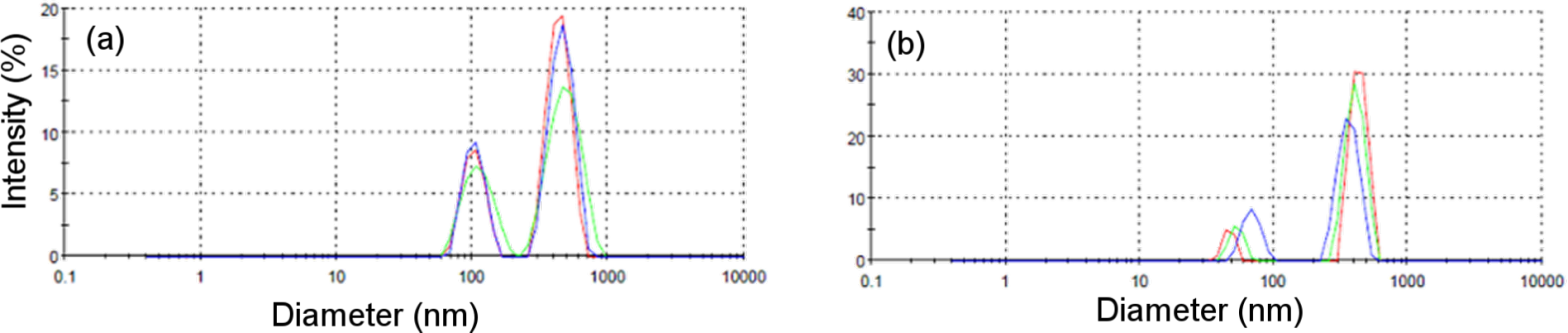
The size distribution profile of (a) Chl*a*–DPPC–MLVs (sample V1) and (b) Chl*a*–Chol–DPPC–MLVs (sample V2).

The formation of DPPC, multilamellar lipid vesicles was confirmed by DLS. The size distribution profile (Figure 2) was bimodal for both types of liposomes.

From these results, one can see that the cholesterol-containing artificial lipid bilayers have smaller dimensions (*Z*_average_ = 567.6 ± 116.2; PDI = 0.322 ± 0.067) than liposomes without cholesterol (*Z*_average_ = 609.8 ± 112.7; PDI = 0.397 ± 0.053).

**Figure 3:**
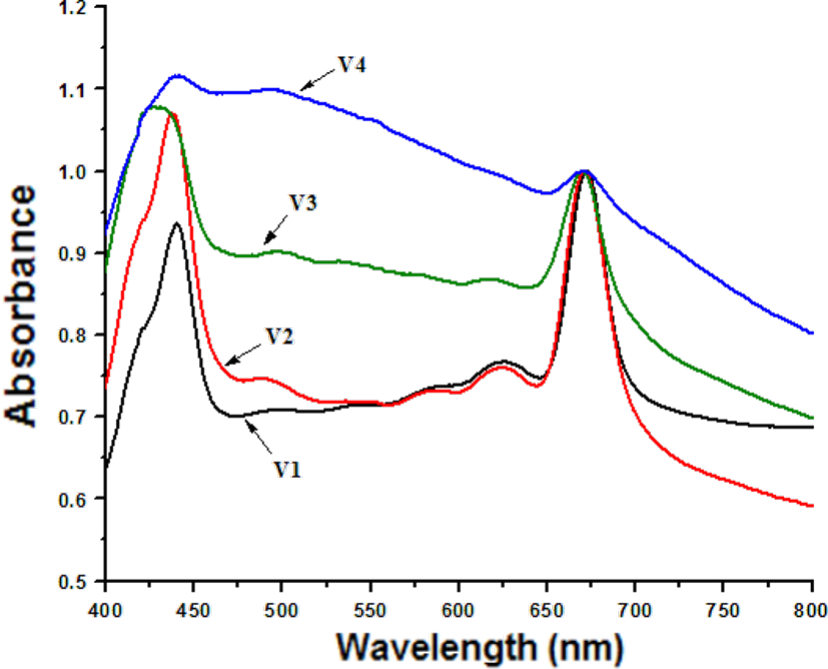
The VIS absorption spectra of Chl*a* in biomimetic membranes and in bio-hybrids.

Chl*a* inserted into the lipid bilayers of liposomes was used as a spectral sensor to monitor the events occurring in the biomimetic membranes. The visible absorption spectra of the samples were normalized versus the absorption at the maximum peak around 670 nm (see Figure 3).

The spectral fingerprint of the Chl*a* sensor consists of specific absorption bands: in the red region (an intense peak at about 670 nm) and in the blue region (Soret band) of the electromagnetic spectrum.

In the case of cholesterol-free samples, the position of the Chl*a* absorption maximum was slightly blue-shifted (from 672 to 670 nm). On the contrary, no change in the location of the Chl*a* peaks in the cholesterol-containing samples (the red absorption band occurred at 671 nm) was observed, because the cholesterol enhances the membrane stability and restricts the lipid mobility [38].

**Figure 4:**
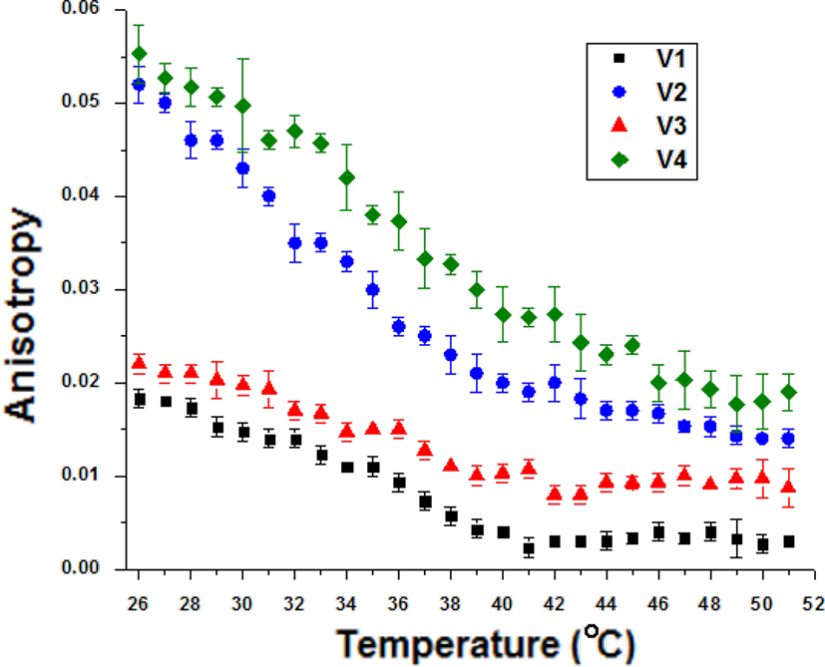
The thermal behavior of the emission anisotropy of Chl*a* in the samples.

**Figure 5:**
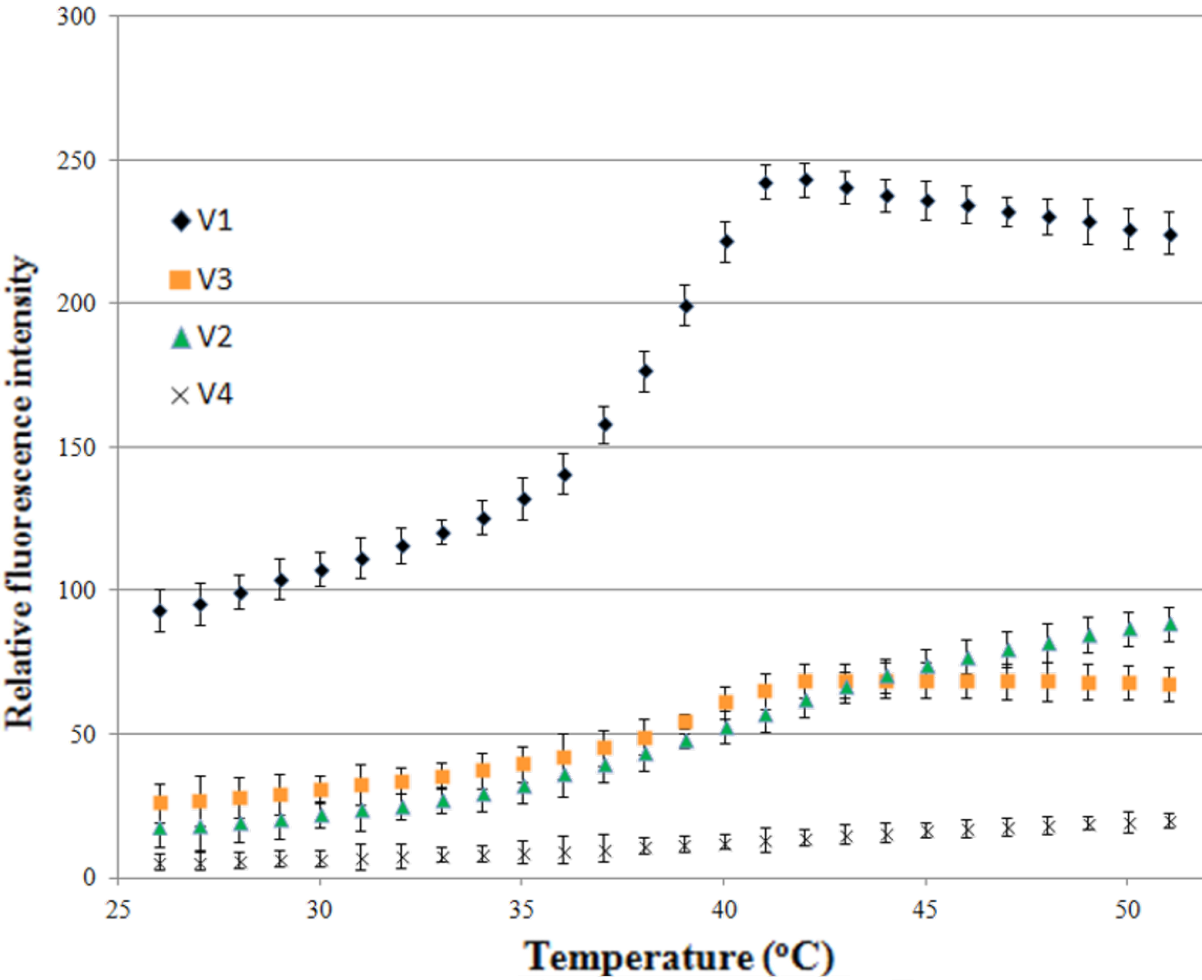
The variation with temperature of the maximum of the relative fluorescence intensity of Chl*a* in the samples.

The architecture of the biomimetic membranes changed after interacting with the carbon nanotubes, evident from the changes in the Soret band. Fluorescence spectroscopy was used in order to gain further information on this interaction at the molecular level. Figure 4 and Figure 5 were obtained by excitation at λ = 430 nm and the emission fluorescence maximum of Chl*a* incorporated in biomimetic membranes and in biohybrids was at 680 nm.

The fluorescence anisotropy of biohybrids is greater than that of liposomes (Figure 4), thus Chl*a* sensed a more rigid environment. This makes Chl*a* beneficial regarding its involvement in non-covalent interactions between lipid vesicles and SWCNTs.

In the case of the biohybrids, the variation with temperature of the maximum of relative fluorescence intensity of Chl*a* embedded in artificial lipid bilayers was much slower as compared with the liposomes alone (see Figure 5). This is due to the presence of carbon nanotubes, which reduces the motion of Chl*a*, which is a result supported by the evolution of the Chl*a* emission anisotropy with temperature. A dramatic quenching of the Chl*a* fluorescence was observed in the presence of SWCNTs due to a more efficient energy transfer between the Chl*a* molecules incorporated in liposomes (ordered along SWCNTs) as a result of interaction with the carbon nanotube sidewall. These findings are in agreement with our previous studies [4–5].

As can be seen in both Figure 4 and Figure 5, the liquid crystal phase of biomimetic membranes (above 41 °C) exhibits low anisotropy and high fluorescence emission intensity due to an increase in the lipid bilayer mobility and hence the chlorophyll has the possibility to move and to minimize the energy transfer leading to fluorescence quenching.

In the gel phase of the artificial lipid bilayers (below 41 °C), high values of anisotropy and low emission fluorescence intensities of Chl*a* inserted into lipid membranes can be observed due to the fluorescence quenching of Chl*a* in a more rigid environment.

The cholesterol-containing samples exhibited high anisotropy and the fluorophore motion is more restricted in this case, that is, Chl*a* senses a more ordered environment. This is due to the presence of cholesterol which induces high order and rigidity in lipid membranes [26,38]. On the other hand, the low fluorescence intensity in the samples with cholesterol could be explained by their small size as compared to the samples without cholesterol (see Figure 2). This leads to fluorescence quenching due to the efficient energy transfer between chlorophyll molecules, which are closer in small vesicles.

### Morphological characterization of biohybrid architectures

**Figure 6:**
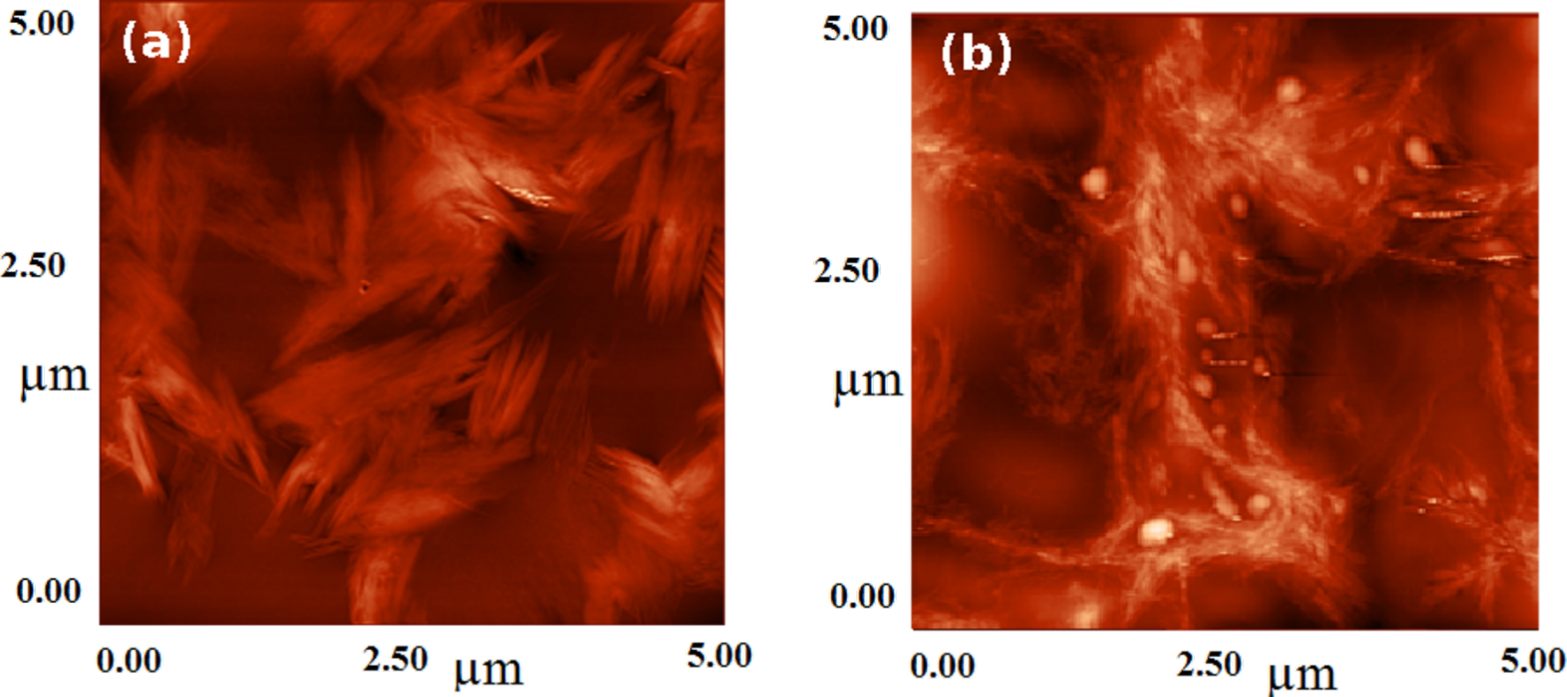
AFM micrographs of nanobioarchitectures without (a) and with (b) cholesterol.

Figure 6 shows a partial exfoliation of CNT bundles in the case of free-cholesterol biohybrids (sample V3). On the contrary, the cholesterol-containing, carbon-based biohybrids (sample V4) proved to be more effective in CNT dispersion. In this case, the AFM analysis revealed a lipid coating around the carbon nanotubes that prevents their aggregation; spherical-shaped profiles of liposomes could be observed along and near the carbon nanotubes. Thus, a carbon nanotube network was formed by the cross-linking of CNTs via liposomes with islands of lipid vesicles. This proves that the bionanocomposite undergoes self-assembly in an ordered fashion (Figure 6b) and not just simple agglomeration of particles (Figure 6a).

### Performance testing of bionanoarchitectures

#### Evaluation of the physical stability of liposome/CNT biohybrids

**Figure 7:**
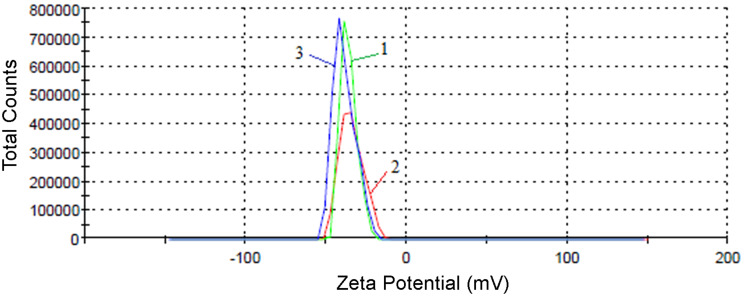
Evaluation of the cholesterol-containing biohybrid (sample V4) stability from the zeta potential distribution, where the ZP was performed in triplicate and the indices 1, 2 and 3 refer to each measurement.

As known from the scientific literature, one of the key factors that determines the physical stability of emulsions and suspensions is the particle charge, which is quantified with the ZP. The ZP is a physical property measured via the electrophoretic mobility of the particles in an electric field [39]. A minimum ZP value of ±30 mV is necessary to ensure the stability of a suspension [40–41]. The physical stability of the biohybrids was rapidly estimated in terms of the ZP. The samples, suspended in PB (pH 7.4) as a biodispersant, carry a negative electric charge. Therefore, if the particles have a large, negative ZP value resulting in high electrostatic repulsion, the dispersion will be stable. The liposomes alone have a low ZP value, thus exhibiting short-term stability, and the repulsive forces are very weak to prevent the particles from coming together. The cholesterol-containing lipid vesicle suspensions, having a ZP value of −19.7 mV, are more stable than vesicles without cholesterol (ZP = −17.2 mV). The carbon-based biohybrids exhibited high stability, possessing a large negative value of the ZP. The cholesterol-containing hybrids were even more stable (ZP = −36.4 mV) than the cholesterol-free biohybrids (ZP = −31.7 mV). Figure 7 presents the stability evaluation of the cholesterol-containing biohybrid (sample V4) by zeta potential distribution. We observed that cholesterol-loaded samples form more stable structures than the samples without cholesterol.

#### The antioxidant behavior of the samples

**Figure 8:**
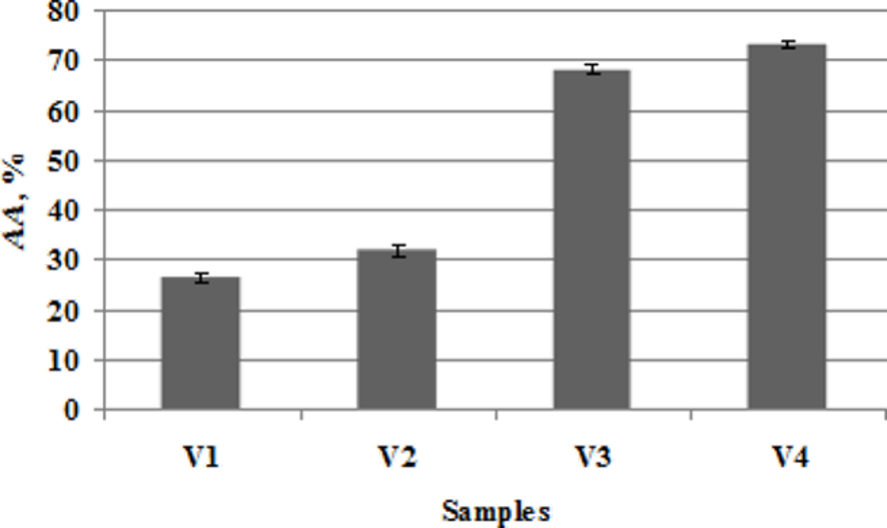
Antioxidant activity of the samples.

In this paper, the chemiluminescence technique was used to estimate the capacity of the SWCNTs/liposomes hybrids to scavenge free radicals. The oxygen radical scavenging ability of the samples was evaluated in terms of antioxidant activity (see Equation 2 in the section Characterization methods). The antioxidant profile of the liposomes and bio-composites displayed in Figure 8 reveals that nanocarbon-based biohybrids possess strong ROS scavenging capacity.

The lipid vesicles alone presented low levels of antioxidant activity (25.9% for V1 and 33.1% for V2), but the addition of SWCNTs to liposomal suspensions resulted in biohybrids with enhanced values of antioxidant activity (68.2% for V3 and 73.25% for V4). The antioxidant behavior of liposomes is due to their chlorophyll content, as it is a photopigment known to possess antioxidant properties [24–25].

One explanation for the good antioxidant action of these SWCNTs/liposomes hybrid systems is that the strong potency of these biohybrids is mainly due to the presence of carbon nanotubes. Although the study of the free-radical scavenging properties of CNTs is an emerging area of nanotechnology, only a few research papers have reported the antioxidant nature of SWCNTs [42–43]. According to some reports [44–45], the free radical scavenger property of CNTs could be attributed to their high electron affinity, suggesting also that ROS may be “grafted” at the CNT surface via radical addition to the nanotube framework. Our previous studies also demonstrated that the presence of SWCNTs in biohybrid materials enhanced their antioxidant properties [4–5,46]. Another more simple explanation is that the chlorophyll embedded in liposomes could convey the antioxidant properties. Finally, the good dispersion state of these hybrids (emphasized by AFM analysis and ZP measurements) could afford more reaction centers that might enhance the capacity of ROS scavenging.

The most potent biohybrids against oxidative attack of free radicals were found to be those with cholesterol (see sample V4), likely due to their better degree of SWCNT dispersibility and their high physical stability as compared to the cholesterol-free biohybrids (sample V3).

#### Antimicrobial activity of samples

The antimicrobial investigations were performed on Gram-negative (*Escherichia coli*) and Gram-positive (*Staphylococcus aureus*, *Enterococcus faecalis*) bacteria. Phosphate buffer solution (pH 7.4) was the negative control for all the samples.

**Figure 9:**
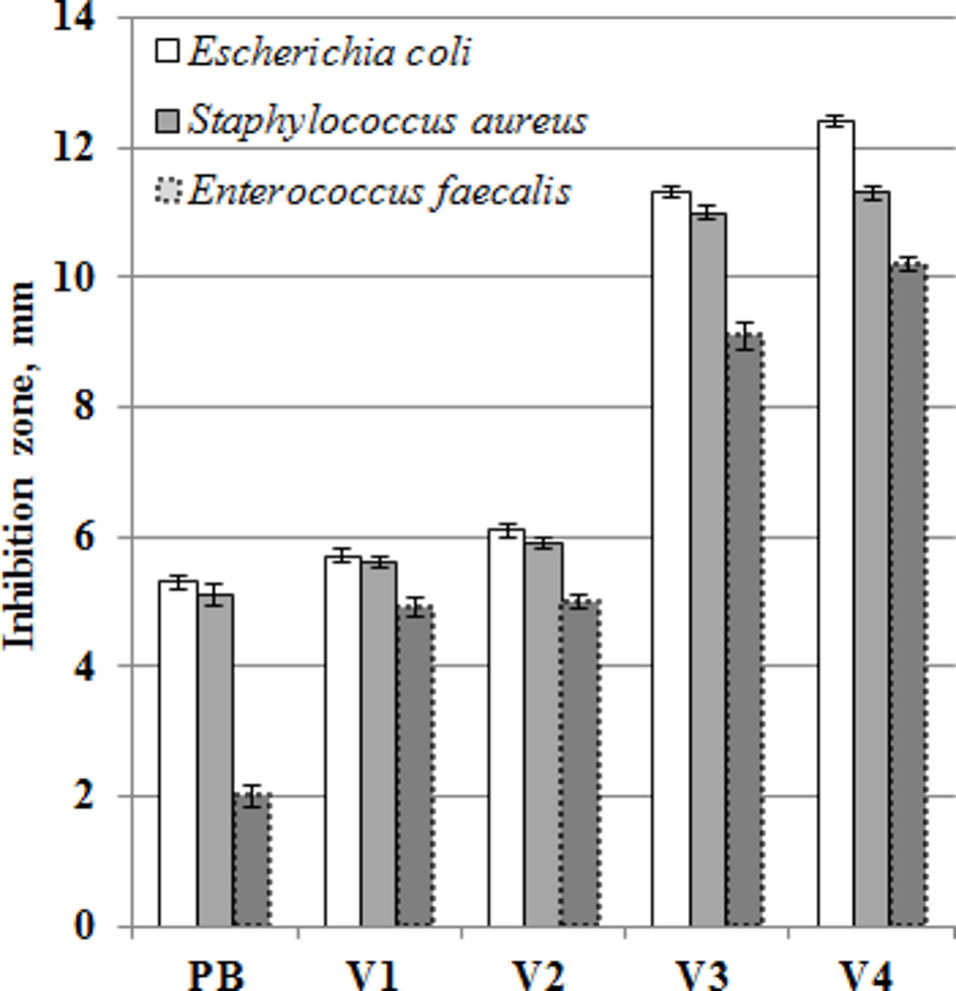
Antibacterial activity of the samples.

The liposomes alone (samples V1 and V2) showed weak antibacterial activity (see Figure 9), offering inhibition zone diameters in the range of 5.0–6.1 mm. However, their biohybrids with carbon nanotubes exhibited enhanced biocidal features due to the presence of SWCNTs, which are known to possess antimicrobial properties [47–48]. Kang et al. [49] pointed out that cell membrane damage resulting from direct contact with carbon nanotubes is the most plausible mechanism leading to bacterial cell death.

Our results showed that small amounts of SWCNTs were enough to achieve high antimicrobial potency (see samples V3 and V4). The cholesterol-free, carbon-based biohybrids (V3) proved to be effective antibacterial materials, presenting inhibition zone diameters of 11.3, 11.0 and 9.1 mm against *Escherichia coli*, *Staphylococcus aureus* and *Enterococcus faecalis*, respectively. On the other hand, the hybrids based on cholesterol-containing biomimetic membranes (V4) have been proven to be the most potent antibacterial material, offering even greater inhibition zones (12.4, 11.3 and 10.2 mm against *Escherichia coli*, *Staphylococcus aureus* and *Enterococcus faecalis*, respectively). An explanation of this behavior of sample V4 compared to sample V3 is a greater degree of dispersibility (according to ZP values and AFM results), which allows for better contact with the bacterial cell. Through this interaction, biomimetic membranes fuse with the natural ones, thus damaging their integrity.

## Conclusion

This paper describes an original design of bionanoarchitectures with interesting properties and potential applications in biomedical and biotechnological fields.

Two types of biohybrids (with and without cholesterol) were developed starting with the chlorophyll-containing, artificial lipid bilayers prepared by hydration of a thin DPPC film. Chlorophyll *a* was successfully used as a spectral marker to obtain information at the molecular level in the artificial lipid bilayers. This enabled spectral monitoring of the bio-based composites by exploiting the fluorescence emission properties and strong visible absorption of the porphyrin macrocycle.

The procedure for obtaining these nanobioarchitectures is simple, efficient, economical (requiring small quantities of raw materials) and eco-friendly. It involves safe, self-assembly steps and ultrasound treatments in a bottom-up approach to build biocidal and antioxidant nanomaterials.

Cholesterol-containing biohybrids were shown to be more stable (ZP = −36.4 mV) and to be the most potent, free radical scavengers, possessing an antioxidant activity value of 73.25%. In addition, the cholesterol-containing, carbon-based biohybrids were the most potent antibacterial materials, offering inhibition zones of 12.4, 11.3 and 10.2 mm in diameter, against *Escherichia coli*, *Staphylococcus aureus* and *Enterococcus faecalis*, respectively. The antibacterial potential of these biohybrids can be exploited in nanotechological applications as antimicrobial coatings.

Based on the fluorescence analysis, we can conclude that the addition of small amounts of carbon nanotubes to liposome suspensions affected the structure and fluidity of the artificial lipid membranes. Chlorophyll *a* sensed the interaction between artificial lipid bilayers and SWCNTs. The cholesterol enhanced the anisotropy, inducing high order in the lipid membranes, and also decreased the mobility in the bilayers.

The bio-coating of CNTs with bio-inspired membranes may be an effective method of increasing the biocompatibility of the CNTs, giving rise to bionanocomposites with good physical stability, having both antioxidant and antimicrobial properties.
